# Water quality and child undernutrition: evidence from 29 low- and middle-income countries and territories

**DOI:** 10.2471/BLT.24.292682

**Published:** 2025-08-25

**Authors:** Dung Duc Le, Long Thanh Giang

**Affiliations:** a Waseda Institute for Advanced Study, Waseda University, Tokyo, Japan.; b Faculty of Economics, National Economics University, 207 Giai Phong Street, Hai Ba Trung District, Hanoi 10000, Viet Nam.

## Abstract

**Objective:**

To determine how *Escherichia coli* contamination of household water affects the probability of stunting and underweight in children younger than 5 years in 29 low- and middle-income countries and territories.

**Methods:**

We used data describing health, nutrition, education, and water, sanitation and hygiene (i.e. *E. coli* testing) from the global Multiple Indicator Cluster Surveys. We conducted multiple linear regression analyses to estimate the effects of *E. coli* contamination on the growth outcomes of stunting and underweight in children, and to explore the underlying mechanisms. We also conducted subgroup analyses to examine heterogeneous effects at both the macro- and microlevels.

**Findings:**

Three quarters of the children in our pooled sample (26 498/35 012) were living in households with drinking water contaminated with *E. coli*. We observed that these children had a 2.3 (95% confidence interval, CI: 0.006 to 0.039) and 1.8 (95% CI: 0.006 to 0.031) percentage point higher probability of experiencing stunting and underweight, respectively, than children living in households with uncontaminated water. Our heterogeneity analyses revealed significant effects of *E. coli* contamination in girls and in poorer households (microlevel); in low- and lower-middle-income countries and territories; and in the World Health Organization African Region and Region of the Americas (macrolevel). Finally, we identified diarrhoea as a potential mechanism through which *E. coli* contamination might adversely affect child growth.

**Conclusion:**

Our findings highlight the critical need to eliminate *E. coli* contamination from household water sources to improve both child health and growth outcomes; changing behaviours related to open defecation remains a key strategy.

## Résumé

**Objectif:**

Déterminer comment la contamination de l’eau des ménages par *Escherichia coli* affecte la probabilité de retard de croissance et d’insuffisance pondérale chez les enfants de moins de 5 ans dans 29 pays et territoires à revenu faible ou intermédiaire.

**Méthodes:**

Nous avons utilisé les données relatives à la santé, à la nutrition, à l’éducation, à l’eau, à l’assainissement et à l’hygiène (c’est-à-dire les tests de détection d’*E. coli*) issues des enquêtes mondiales en grappes à indicateurs multiples. Nous avons effectué des analyses de régression linéaire multiple afin d’estimer les effets de la contamination par *E. coli* sur la croissance infantile (retard de croissance et insuffisance pondérale) et d’étudier les mécanismes sous-jacents. Nous avons également effectué des analyses de sous-groupes afin d’examiner les effets hétérogènes aux niveaux macro et micro.

**Résultats:**

Les trois quarts des enfants de notre échantillon composite (26 498/35 012) vivaient dans des foyers où l’eau potable était contaminée par *E. coli.* Nous avons observé que ces enfants présentaient une probabilité supérieure de 2,3 points (intervalle de confiance (IC) à 95%: 0,006 à 0,039) et 1,8 point (IC à 95%: 0,006 à 0,031) de pourcentage d’être atteint d’un retard de croissance et d’une insuffisance pondérale, respectivement, par rapport aux enfants vivant dans des foyers dont l’eau n’était pas contaminée. Nos analyses d’hétérogénéité ont mis en évidence des effets significatifs de la contamination par *E. coli* chez les filles et dans les ménages les plus pauvres (au niveau micro); dans les pays et territoires à revenu faible et intermédiaire inférieur; et dans les régions d’Afrique et des Amériques de l’Organisation mondiale de la Santé (niveau macro). Enfin, nous avons identifié la diarrhée comme un mécanisme potentiel par lequel la contamination par *E. coli* pourrait nuire à la croissance des enfants.

**Conclusion:**

Nos résultats soulignent la nécessité absolue d’éliminer la contamination par *E. coli* des sources d’eau des ménages afin d’améliorer la santé et la croissance infantiles; le changement des comportements liés à la défécation en plein air reste une stratégie clé.

## Resumen

**Objetivo:**

Determinar cómo la contaminación por *Escherichia* *coli* en el agua de los hogares afecta la probabilidad de retraso en el crecimiento y de bajo peso en niños menores de 5 años en 29 países y territorios de ingresos bajos y medios.

**Métodos:**

Se utilizaron datos sobre salud, nutrición, educación y agua, saneamiento e higiene (incluyendo pruebas de *E.* *coli*) provenientes de las Encuestas de indicadores múltiples por conglomerados. Se realizaron análisis de regresión lineal múltiple para estimar los efectos de la contaminación por *E.* *coli* en los resultados de crecimiento —retraso en el crecimiento y bajo peso— en los niños y para explorar los mecanismos subyacentes. Asimismo, se efectuaron análisis por subgrupos para examinar los efectos heterogéneos tanto a nivel macro como micro.

**Resultados:**

Tres cuartas partes de los niños de la muestra combinada (26 498/35 012) vivían en hogares con agua de consumo contaminada por *E.* *coli*. Se observó que estos niños tenían una probabilidad 2,3 (intervalo de confianza, IC, del 95%: 0,006 a 0,039) y 1,8 (IC del 95%: 0,006 a 0,031) puntos porcentuales mayor de presentar retraso en el crecimiento y bajo peso, respectivamente, que los niños que vivían en hogares con agua no contaminada. Los análisis de heterogeneidad revelaron efectos significativos de la contaminación por *E.* *coli* en niñas y en hogares más pobres (nivel micro), así como en países de ingresos bajos y medios bajos, y en la Región de África y la Región de las Américas de la Organización Mundial de la Salud (nivel macro). Finalmente, se identificó la diarrea como un mecanismo potencial a través del cual la contaminación por *E.* *coli* podría afectar negativamente el crecimiento infantil.

**Conclusión:**

Los hallazgos destacan la necesidad crítica de eliminar la contaminación por *E.* *coli* de las fuentes de agua de los hogares para mejorar tanto la salud infantil como los resultados de crecimiento; el cambio de comportamientos relacionados con la defecación al aire libre sigue siendo una estrategia clave.

## ملخص

الغرض تحديد كيفية تأثير تلوث مياه الشرب المنزلية ببكتيريا الإشريكية القولونية المعوية على احتمالية التقزم ونقص الوزن لدى الأطفال دون سن الخامسة في 29 دولة ومنطقة ذات دخل منخفض ومتوسط.

الطريقة قمنا باستخدام بيانات تصف الصحة، والتغذية، والتعليم، والمياه، والصرف الصحي، والنظافة الصحية (أي اختبار الإشريكية القولونية المعوية) من المسوحات العنقودية متعددة المؤشرات العالمية. قم بإجراء تحليلات تحوف خطية متعددة لتقدير آثار التلوث بالإشريكية القولونية المعوية على نتائج نمو التقزم ونقص الوزن لدى الأطفال، ولاستكشاف الآليات الكامنة وراء ذلك. كما أجرينا تحليلات لمجموعات فرعية لفحص التأثيرات غير المتجانسة على المستويين الكلي والجزئي.

النتائج كان ثلاثة أرباع الأطفال في العين المجمعة لدينا (26498/35012) يعيشون في منازل مياه الشرب بها ملوثة ببكتيريا الإشريكية القولونية المعوية. لاحظنا أن هؤلاء الأطفال لديهم احتمال أعلى بنسبة 2.3 (بفاصل ثقة مقداره 95% (CI): 0.006 إلى 0.039) و1.8 (بفاصل ثقة مقداره 95%: 0.006 إلى 0.031) نقطة مئوية للإصابة بالتقزم ونقص الوزن، على التوالي، من الأطفال الذين يعيشون في أسر ذات مياه غير ملوثة. كشفت تحليلات عدم التجانس لدينا عن آثار كبيرة لتلوث بالبكتيريا الإشريكية القولونية المعوية على الفتيات والأسر الأكثر فقراً (المستوى الجزئي)، وفي الدول والمناطق ذات الدخل المنخفض والمتوسط، والمنطقة الأفريقية ومنطقة الأمريكتين (المستوى الكلي) لمنظمة الصحة العالمية. وأخيرًا، قمنا بتحديد الإسهال كآلية محتملة يمكن أن يؤثر من خلالها تلوث البكتيريا الإشريكية القولونية المعوية بشكل سلبي على نمو الطفل.

الاستنتاج تسلط نتائجنا الضوء على الحاجة الماسة للقضاء على تلوث بالبكتيريا الإشريكية القولونية المعوية من مصادر المياه المنزلية لتحسين صحة الطفل ونتائج النمو؛ ولا يزال تغيير السلوكيات المتعلقة بقضاء الحاجة في العراء استراتيجية رئيسية.

## 摘要

**目的:**

旨在确定在 29 个中低收入国家和地区，大肠杆菌污染家庭用水对 5 岁以下儿童发育迟缓和体重不足概率的影响。

**方法:**

我们使用从全球多指标类集调查中收集的数据来描述健康、营养、教育以及水、环境卫生和个人卫生情况（即大肠杆菌检测）。我们开展了多元线性回归分析，以评估大肠杆菌污染对发育迟缓和体重不足儿童比例增长的影响，并探讨其深层机制。同时，我们还开展了多项亚组分析，以研究在宏观和微观层面的异质化影响。

**结果:**

根据我们的合并样本，有四分之三儿童 (26,498/35,012) 使用的家庭生活用水均已被大肠杆菌污染。据我们观察，与其所用家庭生活用水未被污染的儿童相比，这些儿童出现发育迟缓和体重不足情况的概率分别高出 2.3（95% 置信区间 (CI)：0.006 至 0.039）和 1.8（95% CI：0.006 至 0.031）个百分点。我们的异质性分析结果显示，大肠杆菌污染对女童和较贫困家庭（微观层面）以及低收入和中低收入国家和地区、世卫组织非洲地区和美洲地区（宏观层面）的影响显著。最后，我们确定腹泻是导致大肠杆菌污染可能对儿童发育造成不利影响的潜在机制。

**结论:**

我们的研究结果强调，目前亟需消除大肠杆菌对家庭用水的污染，以改善儿童健康问题和促进儿童发育；改变露天排便相关行为始终是解决该问题的一种关键策略。

## Резюме

**Цель:**

Выяснить, каким образом загрязнение бытовой воды бактерией *Escherichia coli* влияет на вероятность задержки роста и дефицит массы тела у детей младше 5 лет в 29 странах с низким и средним уровнем дохода.

**Методы:**

Авторы использовали данные, описывающие здоровье, питание, снабжение водой, санитарию и гигиену (то есть тестирование на содержание *E. coli*), взятые из глобальных кластерных обследований по множеству показателей. Был проведен множественный линейно-регрессионный анализ для оценки влияния загрязнения *E. coli* на результаты роста в части его задержки и дефицита массы тела у детей и для изучения лежащих в основе этого явления механизмов. Также был проведен анализ в подгруппах с целью изучения гетерогенных эффектов как на макро-, так и на микроуровнях.

**Результаты:**

Три четверти детей в совокупной выборке исследования (26 498 из 35 012) проживали в бытовых условиях, где питьевая вода была заражена бактерией *E. coli*. По наблюдениям авторов, эти дети имели на 2,3 процентных пункта (95%-й доверительный интервал, ДИ: от 0,006 до 0,039) и на 1,8 процентных пункта (95%-й ДИ: от 0,006 до 0,031) более высокую вероятность задержки роста и дефицита массы тела соответственно, нежели дети, которые проживали в бытовых условиях, где вода не была загрязнена. Анализ гетерогенности показал, что загрязнение *E. coli* сильнее сказывалось на девочках и детях из более бедных семей (на микроуровне), в странах и на территориях с низким и низким и средним уровнем дохода и в странах Африканского и Американского регионов ВОЗ (на макроуровне). Наконец, авторы определили, что диарея является потенциальным механизмом, посредством которого загрязнение *E. coli* может негативно влиять на рост детей.

**Вывод:**

Результаты исследования показывают, что устранение загрязнения источников бытовой воды бактерией *E. coli* является критически необходимым условием для улучшения как здоровья детей, так и результатов их роста; изменение поведения в том, что касается открытой дефекации, остается основной стратегией.

## Introduction

Child undernutrition remains a critical global health and development challenge. Although substantial progress has been made in improving child nutrition over the past decades, the prevalence of stunting and underweight among children younger than 5 years remains high in low- and middle-income countries.^1^ Approximately, 149 million children are stunted and 82 million are underweight wordwide.^1^^,^^2^ Early-life undernutrition has detrimental consequences that extend beyond childhood, impairing cognitive development and learning outcomes during childhood, and subsequently reducing economic productivity and earning potential in adulthood.^3^^,^^4^ Leading international organizations have implemented a series of initiatives and commitments aimed at eradicating child undernutrition. The 2030 Agenda for Sustainable Development, with its specific target of reducing child malnutrition (sustainable development goal (SDG) 2.2), provides a strategic framework to guide these efforts.^5^

Emerging research highlights the critical role of the housing environment. Researchers hypothesize that children residing in dwellings with inadequate water, sanitation and hygiene conditions are likely exposed to human or animal faeces containing pathogens with harmful effects on child health and development, potentially contributing to the onset and persistence of childhood undernutrition. This hypothesis is supported by the prevalence of insufficient water, sanitation and hygiene coverage in low- and middle-income countries. Specifically, an estimated one quarter of the global population were not able to access managed drinking water in 2022; of this group, approximately 115 million people were dependent on untreated surface water sources.^6^ Despite these large numbers, several water, sanitation and hygiene programmes in low- and middle-income countries have failed to improve linear growth faltering or other growth outcomes among target children,^7^^–^^10^ making it difficult for policy-makers to justify the role of water, sanitation and hygiene within multisectoral nutrition programmes. Observational studies have yielded mixed findings,^11^^–^^14^ calling for further large-scale research with a focus on the reduction of faecal contamination in the living environment.^15^

We therefore aim to provide internationally comparable estimates of the extent to which *Escherichia coli* contamination of water sources relates to the prevalence of child stunting and underweight in 29 low- and middle-income countries and territories. We utilize *E. coli* testing results at a household level to assess water quality quantitatively, eliminating bias resulting from self-reported improvement in water source or sanitation. We examine the heterogeneous impact of water quality at both the micro- and macrolevel, and explore potential mechanisms through which *E. coli* contamination can influence the likelihood of child stunting and underweight.

## Methods

### Data

We used data from the Multiple Indicator Cluster Surveys (MICS; see also online repository),^16^ a global standardized household survey designed by the United Nations Children’s Fund (UNICEF). Employing a multistage cluster sampling method, MICS probabilistically selects primary sampling units and then randomly selects households within these units. The survey has been collecting information on a wide range of indicators including health, nutrition, education, child protection, water, sanitation and hygiene, providing a holistic perspective of the well-being of children and women in 118 countries and territories since the mid-1990s. Given the objective of this study, we included all countries and territories that collected data on household water tests and child anthropometry. 

To assess faecal contamination, round 6 of MICS incorporated a new module for water quality testing, developed by the World Health Organization (WHO) and UNICEF Joint Monitoring Programme for Water Supply, Sanitation and Hygiene, and which has rigorous quality control measures to ensure test accuracy.^17^ MICS personnel collected 100 mL water samples from selected households at both the point of use and the point of service delivery, and used enzyme substrate nutrient plates (CompactDry™, Nissui Pharmaceutical, Tokyo, Japan) and field-based membrane filtration to measure *E. coli* levels. Laboratory personnel incubated membranes to facilitate *E. coli* growth, and counted *E. coli* colonies after 24 hours. Following WHO guidelines, we created a binary indicator for *E. coli* contamination as determined from a 100 mL water sample (i.e. *E. coli* detected versus *E. coli* not detected).^18^

### Child growth outcomes 

We used the child growth outcomes of child stunting and underweight; child stunting is defined as a height-for-age *Z*-score below two standard deviations (SDs), and child underweight is defined as a weight-for-age *Z*-score below two SDs. We calculated both measures using methods developed for the WHO Multicentre Growth Reference Study.^19^

### Control variables

Following a published study,^20^ we identified a comprehensive set of factors associated with child growth outcomes including characteristics of household heads (age, sex and level of education), parents (age and level of education), children (age and sex) and household size. We provide detailed definitions of these variables in the online repository.^16^ We observed that the correlation between the level of education of the household head and level of education of either parent was relatively low (0.55 for maternal education and 0.65 for paternal education).

### Statistical analysis

We analysed all data using Stata version 17.1 (Statacorp, College Station, United States of America), determining statistical significance at the *P* < 0.05 level. 

#### Descriptive statistics

To describe the study sample, we disaggregated the pooled sample by *E. coli* contamination status, and conducted independent two-sample *t*-tests and *χ*^2^ tests for continuous and categorical variables, respectively.

#### Multiple linear regression

We used a linear probability model to estimate the relationship between water quality and child growth outcomes, while controlling for other variables. A linear probability model is better suited to handle fixed effects, mitigating the problem of incidental parameters associated with probit models.^21^ Further, a linear probability model avoids the potential for bias resulting from mis-specified functional form assumptions (e.g. normal or logistic) inherent in probit or logit models. Because our primary objective is to determine the average association between water quality and child stunting and underweight, rather than predict probabilities, the fact that linear probability models can produce predicted probabilities outside the range of 0–1 is not important.^21^

To mitigate for potential bias, we controlled for multiple levels of fixed effects to account for factors that are common to all individuals, including interviewer, household, primary sampling unit, area of residence (rural versus urban) and administrative regions within individual countries and territories. To capture potential resource allocation disparities between siblings within any household, we also controlled for number of siblings. We clustered standard errors at the household level because this is where each water quality test occurred.^22^ We used original MICS sampling weights for single-country or territory analyses, but rescaled these sampling weights according to country or territory sample size for pooled data analysis. 

Given the potential link between water quality and child health, we also explored whether *E. coli* contamination might indirectly impact child growth by influencing child health outcomes (or mechanisms). We used three indicators of child health to test this hypothesis: cough; diarrhoea; and fever, which were assessed during the two-week period before the survey was conducted. To establish a potential mediating effect, we examined two conditions: (i) *E. coli* contamination as a predictor of child health outcomes; and (ii) the subsequent impact of these outcomes on child growth. 

#### Heterogeneous analysis

To explore potential heterogeneity in the relationship between water quality and child growth outcomes as a result of variations in economic development, household resources and geographic location, we conducted subgroup analyses to identify particularly vulnerable populations. We defined the subgroups at the (i) microlevel: sex of child, household wealth (rich or poor, based on MICS median household wealth index) and whether rural or urban residence; and (ii) macrolevel: World Bank country income classification and WHO region.

### Ethics 

No ethical considerations were applicable because we used publicly available data.^23^

## Results

### Study sample

Our pooled sample for analysis included 35 012 children younger than 5 years. Three quarters of the children (75.7%; 26 498/35 012) were resident in a household in which the drinking water was contaminated with *E. coli*. Of our total sample, 23.1% (8081/35 012) and 12.8% (4484/35 012) were affected by stunting or were underweight, respectively (Table 1). We observed the largest proportion of children living with contaminated drinking water live in Iraq (12.7%; 3376 children). Without considering water contamination status, the largest proportion of those affected by stunting or underweight lived in the Democratic Republic of the Congo (14.3%, 1158 children) or Chad (16.1%; 721 children), respectively (Table 1). 

**Table 1:** Children younger than 5 years either living in households with drinking water contaminated by *Escherichia coli*, stunted or underweight, 29 low- and middle-income countries and territories, 2017–2020

Country or territory; year of survey, by WHO region	Sample size	No. (%)		
		Living in household in which drinking water is contaminated with *E. coli* (*n* = 26 498)	Affected by stunting (*n* = 8081)	Underweight (*n* = 4484)
**African Region**				
Algeria; 2018–2019	1921	678 (2.6)	174 (2.2)	49 (1.1)
Central African Republic; 2018–19	814	736 (2.8)	279 (3.5)	143 (3.2)
Chad; 2019	2442	2428 (9.2)	898 (11.1)	721 (16.1)
Democratic Republic of the Congo; 2017–2018	2571	1925 (7.3)	1158 (14.3)	604 (13.5)
Gambia; 2018	1537	1308 (4.9)	321 (4.0)	222 (5.0)
Ghana; 2017–2018	1300	1061 (4.0)	206 (2.5)	145 (3.2)
Guinea-Bissau; 2018–19	1594	1384 (5.2)	437 (5.4)	233 (5.2)
Lesotho; 2018	204	121 (0.5)	62 (0.8)	18 (0.4)
Madagascar; 2018	1718	1621 (6.1)	698 (8.6)	440 (9.8)
Malawi; 2019–2020	1718	1624 (6.1)	583 (7.2)	206 (4.6)
Sao Tome and Principe; 2019	193	86 (0.3)	26 (0.3)	14 (0.3)
Sierra Leone; 2017	1207	1190 (4.5)	331 (4.1)	162 (3.6)
Togo; 2017	172	162 (0.6)	46 (0.6)	24 (0.5)
Zimbabwe; 2019	612	521 (2.0)	137 (1.7)	55 (1.2)
**Region of the Americas**				
Dominican Republic; 2019	752	614 (2.3)	56 (0.7)	27 (0.6)
Guyana; 2019	497	403 (1.5)	58 (0.7)	34 (0.8)
Honduras; 2019	1922	1357 (5.1)	363 (4.5)	137 (3.1)
Suriname; 2018	421	320 (1.2)	31 (0.4)	31 (0.7)
**South-East Asia Region**				
Bangladesh; 2019	2175	1876 (7.1)	600 (7.4)	477 (10.6)
Nepal; 2019	836	768 (2.9)	266 (3.3)	201 (4.5)
**Eastern Mediterranean Region**				
Iraq; 2018	5465	3376 (12.7)	552 (6.8)	157 (3.5)
Occupied Palestinian territory, including east Jerusalem; 2019–2020	1105	422 (1.6)	78 (1.0)	9 (0.2)
Tunisia; 2018	180	63 (0.2)	18 (0.2)	3 (0.0)
**Western Pacific Region**				
Fiji; 2021	358	200 (0.8)	24 (0.3)	11 (0.3)
Kiribati; 2018–2019	305	290 (1.1)	39 (0.5)	18 (0.4)
Lao People’s Democratic Republic; 2017	1478	1384 (5.2)	510 (6.3)	309 (6.9)
Mongolia; 2018	954	283 (1.1)	95 (1.2)	22 (0.5)
Samoa; 2019–2020	468	209 (0.8)	32 (0.4)	9 (0.2)
Tuvalu; 2019–2020	93	88 (0.3)	3 (0.0)	3 (0.1)

*E. coli: Escherichia coli*; WHO: World Health Organization.

Source: Multiple Indicator Cluster Surveys.

Among children residing in households in which *E. coli* was not detected in the drinking water, the prevalence of child stunting and underweight was 13.5% (1148/8514) and 5.8% (493/8514), respectively. These values are statistically significantly lower (*P* < 0.001) than those measured in households in which *E. coli* was detected in the drinking water, namely 26.2% (6933/26 498) and 15.1% (3988/26 498), respectively. We observed that adults in households in which *E. coli* was not detected in the drinking water were statistically significantly (*P* < 0.001) older and more likely to have a secondary or higher level of education. Households in which *E. coli* was not detected are more likely to have a male household head and be located in urban areas (Table 2).

**Table 2:** Characteristics of children younger than 5 years according to *Escherichia coli* contamination status of household water, 29 low- and middle-income countries or territories, 2017–2020

Characteristic	No. children living in households without or with *E. coli* contamination (%)^a^		Difference, % points	*P*
	Without (*n* = 8 514)	With (*n* = 26 498)		
**Age of child, months (SD)**	29.5 (17.1)	29.2 (17.2)	0.3	0.164
**No. siblings (SD)**	3.1 (2.2)	3.6 (1.8)	−0.5	0.000
**Age of household head, years (SD)**	41.4 (13.2)	40.6 (12.9)	0.8	0.000
**No. in household (SD) **	6.6 (3.9)	7.3 (4.4)	−0.7	0.000
**Age of mother, years (SD)**	30.3 (7.0)	29.2 (6.6)	1.1	0.000
**Stunting**	1 148 (13.5)	6 933 (26.2)	−12.7	0.000
**Underweight**	493 (5.8)	3 988 (15.1)	−9.3	0.000
**Sex of child**				
Male	4 320 (50.7)	13 344 (50.4)	0.3	0.612
Female	4 194 (49.3)	13 154 (49.6)	−0.3	0.542
**Sex of household head**				
Male	7 572 (88.9)	22 969 (86.7)	2.2	0.000
Female	942 (11.1)	3 529 (13.3)	−2.2	0.000
**Education level of household head**				
None	1 092 (12.8)	7 622 (28.8)	−16.0	0.000
Primary	2 031 (23.9)	8 691 (32.8)	−8.9	0.000
Secondary	2 328 (27.3)	6 046 (22.8)	4.5	0.000
Higher	3 063 (36.0)	4 139 (15.6)	20.4	0.000
**Education level of mother**				
None	989 (11.6)	8 095 (30.5)	−18.9	0.000
Primary	1 994 (23.4)	8 342 (31.5)	−8.1	0.000
Secondary	2 298 (27.0)	6 447 (24.3)	2.7	0.000
Higher	3 233 (38.0)	3 614 (13.6)	24.4	0.000
**Education level of father**				
None	626 (7.4)	5 286 (20.0)	−12.6	0.000
Primary	1 765 (20.7)	7 416 (28.0)	−7.3	0.000
Secondary	2 338 (27.5)	6 082 (23.0)	4.5	0.000
Higher	3 785 (44.5)	7 714 (29.0)	15.5	0.000
**Residence**				
Urban	5 332 (62.6)	7 968 (30.1)	32.5	0.000
Rural	3 182 (37.4)	18 530 (69.9)	−32.6	0.000

*E. coli: Escherichia coli*; SD: standard deviation.

^a^ No. (%) presented if not otherwise indicated.

### Multiple linear regression 

We found that children living in households with *E. coli*-contaminated drinking water had a 2.3 (95% confidence interval, CI: 0.006 to 0.039) and 1.8 (95% CI: 0.006 to 0.031) percentage point higher probability of experiencing child stunting and underweight, respectively, relative to children in households with uncontaminated water, holding other factors constant (Table 3). The regression results also revealed similarities in and differences between the determinants of stunting and underweight. Specifically, while age and sex of child, birth order and maternal education were found significantly associated with both outcomes, age and education of household head, along with maternal age, were significant predictors of stunting but not underweight (Table 3).

**Table 3:** Relationships between child or household characteristics and growth outcomes in children younger than 5 years, 29 low- and middle-income countries and territories, 2017–2020

Characteristic	Probability, % (95% CI)	
	Stunting	Underweight
***E. coli* contamination**	2.3 (0.6 to 3.9)	1.8 (0.6 to 3.1)
**Age of child, months**	0.2 (0.2 to 0.2)	0.1 (0.0 to 0.1)
**Sex of child**		
Male	Reference	Reference
Female	−2.7 (−4.2 to −1.3)	−1.5 (−2.5 to −0.5)
**Birth order**	0.5 (0.1 to 1.0)	0.4 (0.1 to 0.7)
**Age of household head, years**	−0.1 (−0.1 to −0.0)	−0.0 (−0.1 to 0.0)
**Sex of household head**		
Female	Reference	Reference
Male	−0.6 (−2.5 to 1.4)	0.5 (−1.4 to 2.3)
**Education level of household head**		
None	Reference	Reference
Primary	−0.2 (−4.0 to 1.0)	0.4 (−1.7 to 2.1)
Secondary	−3.3 (−6.4 to −0.3)	−1.3 (−3.6 to 1.0)
Higher	−4.8 (−7.6 to −2.1)	−2.2 (−4.8 to 0.5)
**Household size**	0.1 (−0.1 to 0.3)	0.0 (−0.2 to 0.2)
**Age of mother, years**	−0.2 (−0.4 to −0.1)	−0.1 (−0.2 to 0.0)
**Education level of mother**		
None	Reference	Reference
Primary	−4.5 (−7.4 to −1.5)	−2.9 (−4.6 to −1.1)
Secondary	−5.1 (−8.4 to −1.9)	−3.4 (−5.2 to −1.7)
Higher	−7.7 (−10.8 to −4.6)	−4.3 (−6.2 to −2.4)
**Education level of father**		
None	Reference	Reference
Primary	−2.0 (−6.1 to 2.0)	−2.1 (−4.4 to 0.1)
Secondary	−1.7 (−5.7 to 2.2)	−1.5 (−4.0 to 1.1)
Higher	−1.8 (−5.2 to 1.6)	−1.4 (−3.8 to 0.9)

CI: confidence interval; *E. coli: Escherichia coli* * *P* < 0.05; ** *P* < 0.01; *** *P* < 0.001.

Multiple linear regression analyses for mechanisms, with child health outcomes as dependent variables and controlling for individual characteristics and fixed effects, revealed that *E. coli* contamination was significantly associated only with the probability of having diarrhoea (1.4; 95% CI: 0.001 to 2.8; Table 4). We subsequently included the diarrhoea variable as an explanatory variable in the main linear regression models with the child growth indicators as outcomes. We observed that diarrhoea significantly predicted child underweight (2.8; 95% CI: 1.4 to 4.3), but its effect on child stunting was not significant (0.8; 95% CI: −0.8 to 2.4; Table 5).

**Table 4:** Relationships between child or household characteristics and health outcomes in children younger than 5 years, 29 low- and middle-income countries and territories, 2017–2020

Characteristic	Probability, % (95% CI)		
	Cough	Diarrhoea	Fever
***E. coli* contamination**	1.5 (−0.4 to 3.4)	1.4 (0.1 to 2.8)	1.03 (−1.1 to 3.1)
**Age of child, months**	−0.1 (−0.1 to −0.1)	−0.2 (−0.3 to −0.2)	−0.8 (−0.1 to −0.0)
**Sex of child**			
Male	Reference	Reference	Reference
Female	−1.0 (−2.6 to 0.6)	−0.2 (−1.3 to 0.9)	−2.1 (−3.4 to −0.9)
**Birth order**	−0.8 (−1.2 to −0.3)	0.1 (−0.2 to 0.3)	−0.2 (−0.6 to 0.2)
**Age of household head, years**	−0.0 (−0.1 to 0.1)	0.0 (−0.0 to 0.1)	0.1 (0.0 to 0.1)
**Sex of household head**			
Female	Reference	Reference	Reference
Male	−0.6 (−2.6 to 1.5)	−0.8 (−2.4 to 0.8)	0.2 (−1.8 to 2.2)
**Education level of household head**			
None	Reference	Reference	Reference
Primary	−0.5 (−3.4 to 2.4)	1.2 (−0.8 to 3.2)	−0.1 (−2.6 to 2.4)
Secondary	−2.2 (−5.3 to 0.9)	2.2 (0.2 to 4.2)	−0.2 (−3.0 to 2.7)
Higher	−1.1 (−5.2 to 2.9)	0.0 (−2.6 to 2.6)	−0.2 (−3.7 to 3.3)
**Household size**	0.1 (−0.2 to 0.3)	−0.0 (−0.3 to 0.2)	−0.1 (−0.4 to 0.2)
**Age of mother, years**	0.1 (−0.1 to 0.2)	−0.1 (−0.2 to −0.1)	−0.0 (−0.1 to 0.1)
**Education level of mother**			
None	Reference	Reference	Reference
Primary	2.2 (−0.1 to 4.5)	0.6 (−1.3 to 2.5)	2.3 (−0.6 to 5.2)
Secondary	0.7 (−2.5 to 3.8)	−0.7 (−2.8 to 1.3)	0.4 (−2.3 to 3.0)
Higher	−2.2 (−5.6 to 1.1)	−1.4 (−3.4 to 0.7)	−1.5 (−4.6 to 1.6)
**Education level of father**			
None	Reference	Reference	Reference
Primary	2.1 (−0.6 to 4.8)	−0.3 (−2.1 to 1.6)	1.7 (−1.5 to 4.8)
Secondary	3.9 (0.7 to 7.0)	−1.1 (−3.5 to 1.4)	−0.9 (−3.6 to 1.7)
Higher	3.0 (0.7 to 5.3)	−0.8 (−2.7 to 1.1)	−0.3 (−2.7 to 2.0)

CI: confidence interval; *E. coli: Escherichia coli*.

**Table 5:** Relationships between child or household characteristics and growth outcomes in children younger than 5 years, when controlling for the health outcome of diarrhoea, 29 low- and middle-income countries and territories, 2017–2020

Characteristic	Probability, % (95% CI)	
	Stunting	Underweight
***E. coli* contamination**	2.3 (0.6 to 3.9)	1.8 (0.5 to 3.1)
**Diarrhoea**	0.8 (−0.8 to 2.4)	2.8 (1.4 to 4.3)
**Age of child, months**	0.2 (0.2 to 0.2)	0.1 (0.1 to 0.1)
**Sex of child**		
Male	Reference	Reference
Female	−2.7 (−4.2 to −1.3)	−1.5 (−2.5 to −0.5)
**Birth order**	0.5 (0.1 to 1.0)	3.4 (0.1 to 0.7)
**Age of household head, years**	−0.1 (−0.1 to −0.0)	−0.0 (−0.1 to 0.0)
**Sex of household head**		
Female	Reference	Reference
Male	−0.6 (−2.5 to 1.4)	0.5 (−1.4 to 2.3)
**Education level of household head**		
None	Reference	Reference
Primary	−1.5 (−4.0 to 1.0)	0.1 (−1.8 to 2.0)
Secondary	−3.4 (−6.4 to −0.3)	−1.4 (−3.7 to 1.0)
Higher	−4.8 (−7.6 to −2.1)	−2.2 (−4.8 to 0.5)
**Household size**	0.1 (−0.1 to 0.3)	0.0 (−0.2 to 0.2)
**Age of mother, years**	−0.2 (−0.4 to −0.1)	−0.1 (−0.2 to 0.0)
**Education level of mother**		
None	Reference	Reference
Primary	−4.5 (−7.4 to −1.5)	−2.9 (−4.6 to −1.2)
Secondary	−5.1 (−8.4 to −1.9)	−3.4 (−5.1 to −1.7)
Higher	−7.7 (−10.8 to −4.6)	−4.3 (−6.1 to −2.4)
**Education level of father**		
None	Reference	Reference
Primary	−2.0 (−6.1 to 2.0)	−2.1 (−4.4 to 0.1)
Secondary	−1.7 (−5.7 to 2.2)	−1.4 (−4.0 to 1.2)
Higher	−1.8 (−5.1 to 1.6)	−1.4 (−3.7 to 0.9)

CI: confidence interval; *E. coli: Escherichia coli*.

### Single-country analysis

The results of a single-country or -territory analysis, available in the online repository,^16^ show that the main contributors to the negative effect of *E. coli* contamination on child stunting estimated from the pooled analysis were Central African Republic, Guinea-Bissau, Sierra Leone, Suriname and Zimbabwe. Similarly, the main contributors to the overall negative effect of *E. coli* contamination on child underweight were Central African Republic, Ghana, Guyana, Kiribati, Lao People’s Democratic Republic, Sao Tome and Principe, and Zimbabwe.

### Heterogeneous analysis

From our subgroup microlevel analyses, we observed that *E. coli* contamination had a strong and significant association with stunting and underweight among girls and children from less affluent households, but no significant associations were observed for boys or children from wealthier households. The impact of *E. coli* contamination on child growth outcomes was found to be similar in rural and urban areas (Table 6).

**Table 6:** Heterogeneous associations of *E. coli* contamination on the probability of stunting or underweight in children younger than 5 years, 29 low- and middle-income countries and territories, 2017–2020

Variable	No. (*n* = 35 012)	Probability, % (95% CI)	
		Stunting	Underweight
**Microlevel variable**			
Sex of child			
Male	17 664	1.2 (−0.7 to 3.1)	1.1 (−0.4 to 2.6)
Female	17 348	3.1 (1.5 to 4.7)	3.2 (1.8 to 4.7)
Household wealth			
Rich	17 356	1.0 (−0.7 to 2.7)	1.0 (−0.6 to 2.7)
Poor	17 656	3.1 (0.7 to 5.6)	3.5 (1.8 to 5.2)
Residence			
Urban	13 300	3.3 (0.5 to 6.1)	1.6 (0.1 to 3.2)
Rural	21 712	3.2 (1.6 to 4.9)	3.1 (1.5 to 4.7)
**Macrolevel variable**			
Country or territory income level			
Low	14 241	4.1 (0.9 to 7.4)	3.3 (0.4 to 6.2)
Lower-middle	15 775	2.9 (0.6 to 5.2)	2.1 (0.6 to 3.6)
Upper-middle	4 996	2.2 (−0.6 to 5.1)	1.5 (−0.6 to 3.5)
WHO region			
African Region	18 003	2.6 (−0.1 to 5.3)	2.9 (0.5 to 5.2)
Region of the Americas	3 592	5.6 (1.5 to 9.7)	−0.0 (−3.2 to 3.1)
South-East Asia Region	3 011	3.3 (−8.5 to 15.0)	−2.2 (−13.7 to 9.4)
Eastern Mediterranean Region	6 750	3.2 (−3.3 to 9.7)	1.4 (−1.9 to 4.7)
Western Pacific Region	3 656	−2.3 (−6.8 to 2.3)	3.2 (−0.1 to 6.5)

CI: confidence interval; *E. coli: Escherichia coli*; MICS: Multiple Indicator Cluster Survey; WHO: World Health Organization.

From our subgroup macrolevel analyses, we observed a significant association between *E. coli* contamination and stunting and underweight among children in low- and lower-middle-income countries or territories, but no significant effect in upper-middle-income countries. We observed significant associations of *E. coli* contamination with child stunting in the Region of the Americas (increased probability of 5.6 percentage points; 95% CI: 1.5 to 9.7; Table 6). Our results also show that the effect of *E. coli* contamination on the probability of underweight was significant in the WHO African Region (increased by 2.9 percentage points; 95% CI: 0.5 to 5.2; Table 6). These heterogeneous results are in accordance with those from our single-country or -territory analysis.

## Discussion

Widely recognized as a human right, access to safe drinking water is a key target of the sustainable development goals (SDG 6.1).^24^ Although investments in water, sanitation and hygiene practices are generally considered crucial for improving child health outcomes in low- and middle-income countries, studies have yielded mixed results regarding the effects of water, sanitation and hygiene practices on child growth outcomes. Our investigation of the extent to which the presence of *E. coli* in household drinking water influences the prevalence of child stunting and underweight has yielded three main findings.

First, our pooled analysis showed that children residing in households with *E. coli* contamination had a higher probability of experiencing child stunting and underweight compared with those in households without *E. coli* contamination. This result is in contrast to previous single-country studies conducted in Nepal and India with similar objectives;^25^^,^^26^ however, these studies were not nationally representative, focused on specific provinces and had relatively small sample sizes. Medical research has documented the association between environmental enteric dysfunction and child growth.^7^^–^^10^^,^^15^ Caused by repeated faecal contamination (e.g. *E. coli*), environmental enteric dysfunction can increase intestinal permeability, leading to reduced nutrient absorption and potential height deficits even without overt diarrhoea or illness. To support this argument, a recent study found a significant association between the presence of *E. coli* in stool samples of children younger than 5 years and child growth outcomes.^27^

Second, we observed that the negative impact of *E. coli* contamination on child growth was mainly driven by its effects on girls, children from less wealthy households, children residing in low- and lower-middle-income countries, and children residing in the WHO African Region and Region of the Americas. A preference for sons, one of the most persistent gender issues in many societies (particularly in low- and middle-income countries), may partially explain the observed disparity between boys and girls. Previous studies have shown that such a preference may affect the allocation of limited household resources such as breastmilk, vitamins, vaccination, protein and health care.^28^^–^^30^ Poverty, a lack of safe drinking water and adequate sanitation, and child growth failure are interconnected. Open defecation is common in many parts of the world, notably in Africa and Asia, which increases the risk of *E. coli* contamination of water sources.^31^ Poor water quality is also a common issue for health-care facilities in low- and middle-income countries, posing a significant infection risk to both patients and personnel.^32^ Therefore, water, sanitation and hygiene practices in health facilities are seen as critical for accelerated progress on maternal and newborn health.^32^

In the WHO African Region, high levels of poverty, widespread open defecation, underdeveloped infrastructure for safe water and sanitation, and inadequate health-care systems are persistent barriers.^6^ In contrast, countries within the Region of the Americas have achieved broader coverage of basic water, sanitation and hygiene services, especially in urban areas. However, stark inequalities persist, with large gaps in income, education and access to good-quality health care.^33^ Reports have indicated that regions with a high prevalence of child growth failure also exhibit the lowest levels of access to adequate water, sanitation and hygiene services.^1^^,^^6^

Third, our finding that diarrhoea serves as a potential pathway through which *E. coli* contamination in water sources may adversely affect child growth complements a recent study. This study, using data from demographic and health surveys across 28 countries, showed that open defecation practices accounted for more than half of the variation in average child height between countries.^31^ Diarrhoeal diseases are typically transmitted through the faecal-oral route; our additional analysis shows that open defecation is positively and significantly associated with the probability of detecting *E. coli* in the water (see online data repository).^16^ Poor water, sanitation and hygiene practices increase an individual’s exposure to faecal pathogens through various pathways including water sources, flies, food and soil, among others.^34^ Diarrhoea is a leading cause of undernutrition among children younger than 5 years, contributing to more than one third of all child mortality cases associated with undernutrition.^34^^,^^35^ According to UNICEF, over 90% of deaths from diarrhoeal illnesses in young children can be attributed to unsafe or inadequate water, sanitation and hygiene practices.^35^

Our study had several strengths. First, by using the results from *E. coli* testing, conducted according to standardized protocols, our measure of water contamination was quantitative and objective. Second, by pooling data from 29 countries and territories across five WHO regions, our findings are more generalizable than single-country studies. Third, by exploring the potential mechanisms through which *E. coli* contamination influences the likelihood of child stunting or underweight, our findings may be of interest to policy-makers. 

Our study also had some limitations. First, although we controlled for a comprehensive set of individual and household characteristics and various fixed effects, variable bias remains a potential concern and prevents any causal inferences. Second, although we acknowledged that there may be water, sanitation and hygiene and malnutrition interventions being implemented in the countries or territories studied, we were not able to consider such interventions. We therefore interpreted our findings as upper bounds of the association of *E. coli* contamination with child growth. Third, the small sample sizes from some of our study countries or territory may have contributed to imprecision in our estimates. Finally, our data structure did not permit the inclusion of country-level factors, such as economic and climate volatility, which might influence water quality and child growth.

Despite these limitations, our findings highlight the critical need to eliminate *E. coli* contamination from household water sources to improve both child health and growth outcomes; changing social norms and behaviours related to open defecation remains a key strategy. The disproportionately negative impacts of *E. coli* contamination on girls, children from poor households and children from low-income countries highlights the need for targeted interventions. To maximize impact, policy-makers should integrate water, sanitation and hygiene interventions, such as household water treatment, improved sanitation to eliminate open defecation and hygiene education, with nutrition-focused strategies, including micronutrient supplementation, promotion of safe food preparation and breastfeeding support. 
